# Strategies for recruitment and retention of adolescent and young adult cancer patients in research studies

**DOI:** 10.1017/cts.2023.669

**Published:** 2023-11-07

**Authors:** Ruixiao Rachel Wang, Julie B. Schweitzer, Samantha Hernandez, Silvia C. Molina, Theresa H.M. Keegan

**Affiliations:** 1 Clinical and Translational Science Center, University of California Davis, Sacramento, CA, USA; 2 Department of Psychiatry and Behavioral Sciences, MIND Institute, University of California Davis, Sacramento, CA, USA; 3 Division of Hematology and Oncology, Center for Oncology Hematology Outcomes Research and Training (COHORT), University of California, Davis Comprehensive Cancer Center, Sacramento, CA, USA

**Keywords:** Adolescent, young adult, cancer, recruitment, retention, clinical trial, longitudinal study

## Abstract

We conducted a literature review to identify commonly used recruitment and retention strategies in research among adolescent and young adult (AYA) cancer survivors 15-39 years of age and examine the effectiveness of these strategies based on the reported recruitment and retention rates. We identified 18 publications published after 2010, including 14 articles describing recruitment strategies and four articles discussing retention strategies and addressing reasons for AYA cancer patients dropping out of the studies. In terms of recruitment, Internet and social networking strategies were used most frequently and resulted in higher participation rates of AYA cancer survivors compared to other conventional methods, such as hospital-based outreach, mailings, and phone calls. In terms of retention, investigators used monetary incentives in all four studies and regular emails in two studies. There was no association between the number of strategies employed and the overall recruitment (*p* = 0.09) and retention rates *(p* = 0.33). Future research and planned studies testing recruitment and retention strategies are needed to identify optimal, modern communication procedures to increase AYA participation and adherence. More education should be provided to AYAs to increase their knowledge of research studies and strengthen the connection between AYA cancer survivors and their health providers.

## Introduction

Adolescent and young adult (AYA) patients aged 15–39 years are recognized as a unique population within the oncology community. Worldwide, more than 1.2 million AYAs are diagnosed with cancer annually, and nearly 90,000 AYAs were diagnosed in 2020 in the United States [1], yet there is a paucity of studies specifically targeting AYAs [2]. AYA participation in clinical and longitudinal research studies is needed to advance diagnostic and interventions to improve AYA cancer survivors’ length and quality of life. There may be inherent advantages in participating in studies for AYA patients, given that AYAs who participated in clinical trials had a lower risk of mortality and had a higher overall survival rate [3]. However, AYA patients are less likely to participate and remain in research studies, likely due to loss of contact with oncology centers when adolescents reach the age of majority (and thus, the parent is no longer legally the person of contact), as well as their developmental stage and lifestyle factors [4] that may not be considered by the investigators [5]. As a result, recruitment and retention rates among AYAs in studies are significantly lower than in studies targeting patients at younger and older developmental stages. Data from the United States, United Kingdom, Italy, and Australia suggest that the AYA group has the lowest clinical trial participation rate [6]. Therefore, it is crucial to develop effective strategies to enroll and retain AYA cancer patients in clinical and longitudinal studies.

The Internet has become the mainstream platform for acquiring and disseminating information. Digital tools, such as social media and email, play an important role in recruiting and retaining participants. We hypothesized that an increasing number of strategies used to recruit/retain AYA cancer survivors would be associated with higher recruitment and retention rates. Therefore, we conducted a literature review to identify commonly used recruitment and retention strategies in research among AYA cancer survivors and examined the effectiveness of these strategies based on reported recruitment and retention rates.

## Materials and Methods

We used PubMed and Google Scholar to identify existing studies and reviews on AYA recruitment and retention methods for longitudinal research and clinical trials in oncology. Considering the rapid development of the Internet in the past ten years, results were restricted to publications no earlier than 2010 to review more current research. We included only studies published in English.

To narrow the publications in cancer-specific research, keywords of “cancer,” “AYA,” “adolescent,” “young adult,” “recruitment,” “retention,” “participation,” “rate,” and “strategy” were used. These keywords were combined multiple times as “adolescents cancer recruitment rate,” “young adult cancer recruitment rate,” “adolescent cancer retention rate,” “young adult cancer retention rate,” “AYA recruitment and retention strategy,” “adolescent and young adult cancer participation,” and “AYA cancer recruitment and retention” to get a comprehensive search of relevant studies. Additionally, citations of the selected articles, especially systematic reviews, were evaluated and filtered with the same inclusion and exclusion criteria so that studies missed in the keyword searching stage could be included. Studies that were not cancer-specific, did not target AYAs, or did not specify a population age range were excluded. A total of 10 articles were excluded, including 5 articles without a description of recruitment and retention strategies.

A Spearman correlation test assessed the association between several strategies used in each study and overall recruitment and retention rates. A *p*-value of < 0.05 was considered statistically significant.

## Results

The final search yielded 18 publications (Table 1). Fourteen articles described recruitment strategies, and four articles discussed retention strategies and addressed reasons for AYA cancer patients dropping out from the studies.

**Table 1.** Summary of included studies of adolescent and young adult (AYA) cancer survivors

Study	Study type	Sample size	Cancer type	Main focus
Juraschek *et al*. 2018 [8]	Original research – clinical trial	406	Non-specified types of cancer survivors	Discusses the use and cost-effectiveness of online recruitment strategies compared to traditional forms of recruitment
Rabin *et al*. 2012 [7]	Original research – clinical trial	802	Non-specified types of cancer survivors	Reports a wide range of recruitment strategies used for a web-based physical activity intervention
Casillas *et al*. 2019 [33]	Original research – clinical trial	269	Non-specified types of childhood cancer survivors	Compares the abilities of a text-messaging system and a peer navigator program to reach out to AYA
Ulrich *et al*. 2012 [14]	Original research – clinical trial	32	Cancer of any type (female breast and/or ovarian, colorectal, prostate, Hodgkin and non-Hodgkin lymphoma, etc.)	Discusses benefits and burdens of research participants in cancer clinical trials
Cantrell *et al*. 2012 [4]	Original research – clinical trial	N/A	Non-specified type of childhood cancer survivors	Describes the challenges to recruit and retain female AYA childhood cancer survivors in longitudinal research
Benedict *et al*. 2019 [9]	Original research – surveys and interviews	435	Non-specified types of cancer survivors	Compares hospital-based and social media recruitment strategies and evaluates group differences in patient
Clinton-McHarg *et al*. 2011 [34]	Original research – cross-sectional study	411	Lymphoma, melanoma of the skin, thyroid, testicular cancer, etc.	Describes recruitment rates for AYA recruited through a cancer registry
Hendricks-Ferguson *et al*. 2013 [19]	Original research – clinical trial	226	Oncology, patients undergoing a hematopoietic stem cell transplant	Overview of factors related to AYA recruitment and reasons for refusal
Hagström *et al*. 2020 [35]	Original research – clinical trial	213	Non-specified types of cancer survivors	Investigates the feasibility and efficacy of cognitive-behavioral therapy for AYA cancer survivors
Gorman *et al*. 2014 [10]	Four original research studies – focus groups, cross-sectional and cohort studies	534 (recruited)	Breast cancer, lymphoma, leukemia, thyroid, soft tissue cancers, brain cancer, etc.	Describes recruitment strategies used for young adult female cancer survivors
Seltzer *et al*. 2014 [11]	Original research – cross-sectional study	60	Non-specified childhood cancer	Reports childhood cancer survivors’ use of social networking site for recruitment of survivorship research
Le *et al*. 2017 [16]	Original research – clinical trial	19 (enrolled)	Non-specified childhood cancer	Reports a pilot study of a home-based exercise intervention with a motivational activity tracker
Rosenberg *et al*. 2016 [18]	Original research –cohort study	57	Non-Central-Nervous System cancer with chemotherapy treatment prior to enrollment	Assesses the feasibility of qualitative methods to improve retention
Valle *et al*. 2013 [36]	Original research – clinical trial	167	Hematologic cancer, breast cancer, head and neck cancer, etc.	Tests the physical activity interventions to improve health and quality of life among AYA cancer survivors
Valle *et al*. 2022 [12]	Original research – clinical trial	280	Breast cancer, Hodgkin’s lymphoma, melanoma, thyroid cancers, etc.	Describes recruitment strategies in a randomized controlled trial of a mobile health physical activity intervention
Vlooswijk *et al*. 2022 [15]	Original research – cross-sectional study	4,010	Breast cancer, germ cell tumors, lymphoid hematological malignancies, etc.	Examined the effect of invitation methods on response rates and non-participation in AYA cancer survivor subgroups
Hulbert-Williams *et al*. 2019 [13]	Original research – cohort study	200	Breast cancer, colorectal cancer, gynecological cancers, etc.	Compares the effectiveness of recruiting cancer survivors through various social media
Taylor *et al*. 2017 [17]	Original research – cohort study	10	Hodgkin lymphoma, osteosarcoma, acute myeloid leukemia, testicular, brain, and thyroid cancer	Describes retention strategies in a longitudinal study examining AYA’s view toward continuing study participation

N/A = not available.

### Recruitment strategies

A total of 12 methods were used to recruit potential participants (Table 2). Internet-based (*n* = 10) and hospital-based (*n* = 6) strategies were the primary approaches used to recruit participants. Of the 14 studies that report recruitment methods, 64.3% (*n* = 9) reported using financial incentives, ranging from $20 to $50 per person.

**Table 2.** Recruitment strategies used in each study of adolescent and young adult cancer survivors

Strategies	Juraschek *et al*. 2018 [8]	Rabin *et al*. 2013 [7]	Casillas *et al*. 2019 [33]	Ulrich *et al*. 2012 [14]	Cantrell *et al*. 2012 [4]	Benedict *et al*. 2019 [9]	Clinton-McHarg *et al*. 2011 [34]	Hagström *et al*. 2020 [35]	Gorman *et al*. 2014 [10]	Seltzer *et al*. 2014 [11]	Valle *et al*. 2013 [36]	Valle *et al*. 2022 [12]	Vlooswijk *et al*. 2022 [15]	Hulbert-Williams *et al*. 2019 [13]	Total
Community organizations	✓								✓		✓	✓			4
Recruitment at clinics	✓	✓		✓		✓			✓			✓			6
In-person cancer-related events		✓													1
Direct mail	✓	✓	✓					✓				✓	✓		6
Internet and social media	✓	✓	✓		✓	✓			✓	✓	✓	✓		✓	10
Advertisements in periodicals	✓														1
Phone calls		✓						✓				✓			3
Text messaging			✓												1
Word of mouth	✓	✓							✓						3
Brochures/flyers	✓	✓			✓						✓	✓			5
Broadcasting shows/radio shows		✓			✓		✓								3
Advertisement at conferences												✓			1
Total	7	8	3	1	3	2	1	2	4	1	3	7	1	1	44

### Internet and social networking

**Figure 1.**
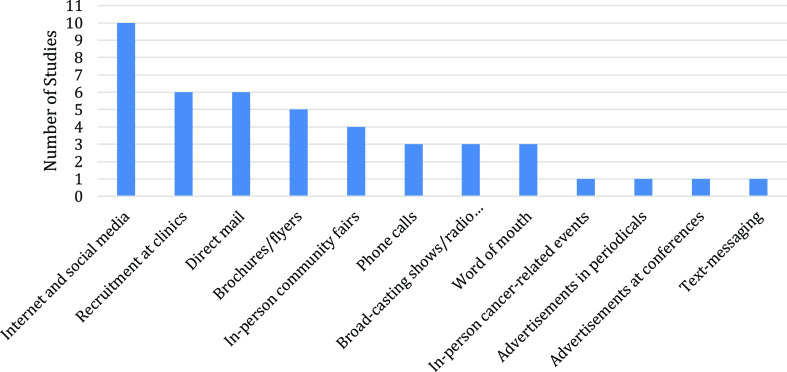
The number of studies with adolescent and young adult cancer survivors using each recruitment strategy.

Among the 14 studies, 10 used recruitment strategies through the Internet and social networking sites (Fig. 1). Rabin et al. [7] recruited participants via social media through organizations that serve the needs of cancer survivors and on multiple websites, including Craigslist. However, recruiting through mailings and approaching patients in person in oncology clinics were the most productive strategies [7]. In contrast, the other 10 studies demonstrated that Internet-based outreach was more effective than conventional strategies (e.g., in-person recruitment at clinics and phone calls). Juraschek *et al*. [8] used paid banner advertisements on Facebook to attract users to the trial’s website. The advertisement was designed to stay on the screen for the whole session. English language users within the targeted age range with selected Facebook interests were shown in the advertisement. Facebook recorded over three million impressions to 124,476 people and 4,410 clicks on the advertisement, resulting in 24 respondents and four participants.

After failing to recruit the expected number of participants at the three designated oncology centers, Cantrell *et al*. [4] employed alternative strategies of which online methods yielding the most enrollment (80%, *n* = 128/160) of participants. These approaches included emails sent by the directors of cancer survivorship organizations, posting on cancer survivorship organizations’ websites, Facebook paid advertisements, and Facebook posts on cancer survivorship sites. Similarly, Benedict *et al*. [9] indicated a higher participation rate (37%, *n* = 54/146) achieved through social media recruitment. Facebook and Instagram were used to post descriptions of the study and contact information.

Gorman *et al*. [10] found that social media and Internet-based strategies resulted in the highest enrollment rate nationally. Locally, they deployed advertisements of the studies in online university-based newspapers, a local Craigslist website, and the cancer center webpage; young cancer survivors were also reached out via email and Facebook. Nationally, AYA cancer survivor organizations posted information on Facebook and Twitter. The research team also developed a Facebook page for this study, and they placed advertisements on Craigslist websites in large cities across the USA. Lastly, Seltzer *et al*. [11] conducted a pilot study to interview childhood cancer survivors on their opinions on using social networking sites as a recruitment strategy, of which 79% of respondents expressed a positive attitude [11].

Valle *et al*. [12] recruited 11.7% more participants through social media (52.4%), including unpaid Facebook posts (45%), paid advertisements (5.9%), and Twitter posts (1.5%), than through direct mailings (40.7%). Among the participants, females were more likely to be recruited through social media, while males were more likely to be recruited by direct mailing. Those recruited through social media were more likely to have a college degree [12]. Similarly, in a secondary analysis from an international cohort study, Hulbert-Williams *et al*. [13] found that paid Facebook posting, Twitter advertisements, and Reddit posting were the most cost-effective recruitment methods, which respectively yielded 27%, 32%, and 22%.

### Clinic/cancer center-based recruitment

Another widely used recruitment strategy was done through hospitals, oncology clinics, and cancer centers. AYA cancer survivors were contacted either by their physicians in person or by the investigators of the study, given the approval of their oncology providers.[7,9,10,14] Ulrich *et al*. [14] gained access to eligible cancer survivors through research nurses and physicians, and the principal investigator received information on potential participants monthly via the clinical research unit of the cancer center. With the approval of the cancer center, the investigators then contacted cancer survivors in person at the clinic or through phone calls. Benedict *et al*. [9] selected potential participants by evaluating electronic medical records. Upon the agreement of oncology providers, eligible cancer survivors were mailed the invitation for the study, and follow-up phone calls were made to confirm eligibility and obtain informed consent. Gorman *et al*. [10] also contacted the university-affiliated hospital and health providers to hand out study flyers and postcards to patients.

### Other recruitment strategies

Other recruitment strategies included direct postal mailings (*n* = 4 studies), broadcasting and radio shows (*n* = 3), word of mouth (*n* = 3), brochures and flyers (*n* = 3), phone calls to eligible AYA cancer survivors (*n* = 2), and in-person community fairs (*n* = 2). In-person cancer-related events, advertisements in periodicals, text messaging, and outreach via university health centers were less frequently used (*n* = 1). Notably, Valle *et al*. [12] concluded that direct mailing, compared with recruiting through a health registry or community/conferences, was the most cost-effective recruitment strategy and resulted in the highest participation rate.

### Recruitment Rate

The overall recruitment rate could be identified in six out of the 14 studies, with five rates reported by the original studies and one calculated from available data (Table 3). Juraschek *et al*. [8] did not state the rate, although they included the number of participants *(n* = 406) and number of people who were shown the Facebook advertisements (*n* = 124,476). Benedict *et al*. [9] reported an enrollment rate from social media (37%; *n* = 54/146) and an enrollment rate from hospital-based recruitment (7%; *n* = 21/289). The overall recruitment rate (17%) was calculated using the total number of enrollees divided by the potential participants. We did not observe an association between the number of recruitment strategies and the recruitment rate (*p* = 0.092).

**Table 3.** Recruitment rates in studies of adolescent and young adult cancer survivors

Strategies	Juraschek *et al*. 2018 [8]	Rabin *et al*. 2013 [7]	Casillas *et al*. 2019 [33]	Ulrich *et al*. 2012 [14]	Cantrell *et al*. 2012 [4]	Benedict *et al*. 2019 [9]	Clinton-McHarg *et al*. 2011 [34]	Hagström *et al*. 2020 [35]	Gorman *et al*. 2014 [10]	Seltzer *et al*. 2014 [11]	Valle *et al*. 2013 [36]	Valle *et al*. 2022 [12]	Vlooswijk *et al*. 2022 [15]	Hulbert-Williams *et al*. 2019 [13]
Total number of strategies used	7	8	3	1	3	2	1	2	4	1	3	7	1	1
Recruitment Rate	N/A	N/A	32%	N/A	N/A	17%	50%	4.7%	N/A	79%	N/A	N/A	36%	N/A

N/A = not available.

Including paper questionnaires and sending reminders increases the recruitment rate of AYA cancer survivors [15]. In a cross-sectional study, Vlooswijk *et al*. [15] divided the target population into three different categories with different invitation strategies – paper-optional questionnaire with reminders sent, paper-optional questionnaire without reminders, and paper-included questionnaire with reminders. The invitation letters were sent with a link to the online questionnaire, online consent form, and a pre-stamped envelope. The paper-optional group was provided with guides to request a paper version of the questionnaire, whereas the paper-included group was mailed with the paper version directly. The paper-included group resulted in the highest recruitment rate (41%, *n* = 544), and the no-reminder group resulted in the lowest rate (26%, *n* = 429), and the rate was not reported for the paper-optional group [15].

### Retention strategies

Among the identified studies, only four discussed retention strategies and three provided a retention rate (Table 4). Cantrell *et al*. [4], Le *et al*. [16], and Taylor *et al*. [17] reported retention rates of 61%, 79%, and 58%, respectively. Among these studies, we did not observe an association between the number of retention strategies and the retention rate (*p* = 0.333).

**Table 4.** Retention strategies in studies of adolescent and young adult (AYA) cancer survivors

Strategies	Rosenberg *et al*. 2016 [18]	Cantrell *et al*. 2012 [4]	Le *et al*. 2017 [16]	Taylor *et al*. 2017 [17]	Total
Regular emails	✓	✓			2
Incentives	✓	✓	✓	✓	4
Participants wearing a motivational activity tracker			✓		1
Request for updated information				✓	1
AYA branded the study with logo				✓	1
Regular newsletters and postcards with latest findings				✓	1
Showing photos of research team				✓	1
The commercial research organization that administers the survey sends out letters for reminders				✓	1
Having option of online or telephone interviews after the first data collection				✓	1
Obtaining stable contact details at the initial survey/interview				✓	1
Flexibility in continuing participation				✓	1
Thank you letters after each round of data collection				✓	1
Having a study dedicated phone number				✓	1
Total	2	2	2	11	17
Retention rate	N/A	61%	79%	58%	

N/A = not available.

In the study conducted by Rosenberg *et al*. [18], a group of participants were invited for interview and survey completion, and the other group was only invited to take surveys. Surveys were given at three time points spread over 18 months. Participants involved in interviews had greater completion of the surveys (98% completion rate), compared with survey-only participants (58% completion rate). Besides sending regular email reminders during the 18-month period, the investigators found additional monetary incentives (value not specified) and interview appointments increased the retention rate due to a sense of obligation. In terms of surveys, more AYA cancer patients preferred paper over online versions. Similarly, in the second study by Cantrell *et al*. [4], participants were also offered monetary incentives (value not specified), and they were reminded via emails throughout the duration of the study, which resulted in a 61% retention rate.

In the third study, Le *et al*. [16] conducted a 6-month physical activity intervention using Fitbit One devices. Eligible AYA cancer survivors were required to wear the tracker daily for six months. Participants were offered $10 gift cards per month for wearing the tracker for more than 20 days; another $20 was awarded for completing the baseline evaluations and an additional $20 for follow-up evaluations. This resulted in an overall retention rate of 79% [16]. Lastly, Taylor *et al*. [17] reported that the retention rate increased from 30% in the third round of data collection to 58% among AYA cancer survivors by implementing a variety of retention strategies: regularly updating study news to the participants, sending frequent email reminders of events, sending postcards and small gifts to participants to express gratitude, providing staff contact information to participants, having multiple options for data collection, obtaining stable contact details from participants, giving certificates of enrollment upon completion of study components, and providing a phone number for participants to contact study staff.

### Reasons for dropout

**Figure 2.**
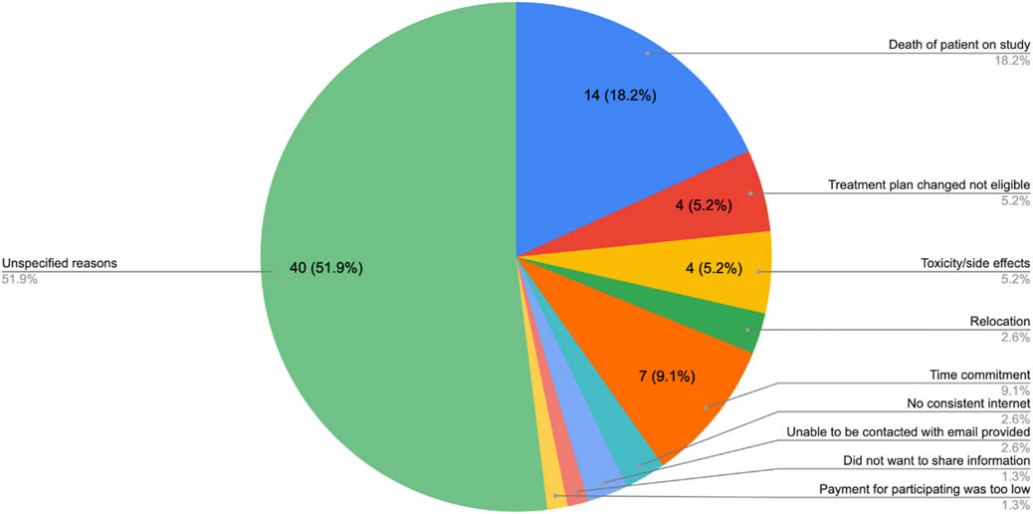
Reasons for participants dropping out of the studies.

Three articles discussed the reasons for participants dropping out from studies, including two clinical trials and a survey-based study (Fig. 2) [4,18,19]. In addition to the death of patients and change in eligibility, too much time commitment, side effects, and relocation were the top reasons for dropping out. Cantrell *et al*. [4] identified that control groups were more likely to result in a higher attrition rate (55%), as the AYA cancer survivors assumed they would be assigned to the intervention group.

## Discussion

Internet-based outreach to AYA cancer survivors became a common strategy after 2010 based on 14 published studies that included information on recruitment methods. Despite that most studies did not provide the recruitment rates of each strategy, studies generally reported a higher participation rate resulting from Internet and social networking recruitment compared to recruitment at oncology clinics and cancer centers, supporting our hypothesis that the use of Internet-based outreach would increase recruitment rates of AYA cancer survivors. In terms of retention strategies, much of the existing research addresses the attrition rate among AYA cancer survivor studies, with little published literature on methods to improve retention rates. The most utilized retention method was monetary incentives of cash and gift cards, which was mentioned in all four studies, followed by regular emails to participants used in two studies. No studies used Internet-based strategies to increase retention rates, identifying an important area to consider in future studies. To advance the field of research in AYA cancer survivors, investigators should report their recruitment and retention rates and strategies in all publications reporting their study methods.

Studies conducted across broader age groups could offer recruitment strategies for AYA cancer survivors. After evaluating 68 studies across all ages on strategies to improve recruitment in randomized trials from different countries, Treweek *et al*. [20] found that informing participants of what they will receive in the trial and phoning people who did not respond to the invitation were effective in improving the recruitment rate. In a systematic review of recruitment strategies used to enroll cancer survivors of all ages with organ failure into clinical trials, Boland *et al*. [21] found that cancer survivors who responded through the cancer registry were less likely to consent to participate than those who responded to local media advertisements. This finding is expected, as cancer registries include all patients diagnosed with cancer, while those who respond to local media advertisements represent a subset of cancer patients with more interest in participating in research. On the other hand, Beckie *et al*. [22] reported that the highest recruitment rate (73%, *n* = 184) was achieved from automatic hospital referral to the cardiac rehabilitation clinical trial, although a wide range of recruitment strategies other than referrals were employed, including mailings, media advertisements, and community outreach.

In our review, we did not observe a clear association between the number of strategies employed and the overall recruitment and retention rates. Our assessment was limited by the availability of data, as among the 18 studies identified for both recruitment and retention strategies, only five of them provided information on their recruitment rate, one of them indicated the number of enrollees and potential participants for us to calculate the recruitment rate, and three articles reported their retention rate. However, prior studies not restricted to AYA cancer survivors have found conflicting results. After conducting a systematic review of 88 studies on 985 retention strategies, Robinson *et al*. [23] found a small, positive correlation between the number of strategies used and the overall retention rate (r = 0.24, *P* = 0.027). In contrast, Teague *et al*. [24] claimed that the total number of strategies was not related to retention in a meta-analysis of 143 longitudinal cohort studies. It may be that specific strategies, rather than the number of strategies, impact retention rates. Some commonly used retention strategies include obtaining multiple contacts for each participant and enlisting the cooperation of family and friends (28% of the abstracted studies), offering flexible clinic appointment hours and locations (15%), and assigning one primary clinician to each participant (10%) [19]. Some emerging strategies included using social media and text messaging to trace participants, as well as managing study websites and social media to update study news and events with the participants [23,24]. These strategies may also be transferable to AYA cancer survivors.

According to the three studies that described why AYA cancer survivors withdrew from studies, the most common reasons were concerns about the time commitment, side effects, and relocation. In addition, Roick *et al*. [25] found that participants of all ages with less education were more likely to withdraw from a randomized clinical trial, which may relate to less of an understanding of the benefits of research trials and the importance of participation and retention in the trial. Buchanan *et al*. [26] also found that adolescents lacked knowledge about clinical trials – misinformation and lack of awareness resulted in poor readability and clarity of consent, which negatively affected participation and commitment to the study. Unfortunately, increasing education of clinical trials was not found to improve recruitment rates for AYA cancer survivors aged 18–24 years old [26]. However, several publications demonstrated that health providers’ understanding of clinical trials affected patients’ awareness. AYA cancer survivors were more willing to participate and stay in the clinical trial if they received information from their physicians [27–29]. Therefore, educating investigators on the importance of providing clinicians with study materials that clearly explain the type and importance of continued research participation in language easily accessible to AYAs could improve retention. Additionally, cancer survivors with lower income and at risk of poverty were more likely to leave the study compared to middle-income patients [25], highlighting potential financial barriers to participation.

Furthermore, Buchanan *et al*. [26] also discussed psychosocial barriers for AYA cancer survivors to enroll and remain in clinical trials. Compared to children, AYAs have higher anxiety levels about their cancer diagnosis, especially when facing changes in appearance and autonomy as a result of cancer and treatments. AYAs expressed concerns regarding reduced quality of life, loss of autonomy, and adverse effects of enrollment in clinical trials. Medical mistrust is another barrier preventing them from participating in studies, especially among racial/ethnic minority patients [26].

There are also system-level barriers that hinder the recruitment and retention of AYA cancer survivors into studies. Compared to cancer survivors < 15 years of age who receive care in pediatric oncology facilities, adolescent cancer survivors have lower participation rates in clinical trials [6]. Enrollment rates vary by age, cancer type, treatment setting, race/ethnicity, and health insurance [30]. AYA cancer survivors treated in adult oncology facilities have more limited access to trials compared to AYA treated in pediatric oncology settings. A prior study showed that the place of treatment impacts the trial participation rate. Patients aged 10–19 years had a 35% higher rate of enrollment into clinical trials if being treated in a pediatric compared to the adult oncology setting [31]. A qualitative study also identified that poor communication between pediatric and adult oncologists could prevent AYAs from enrolling in clinical trials [32]. In addition, lack of health insurance for AYAs, lack of awareness of open clinical trials, strict eligibility criteria, and arbitrary age cutoffs of trials could also contribute to the low participation rate of clinical trials for AYA cancer survivors [6,30,32]. Because most AYA cancer survivors are treated in the community setting, access to clinical trials may be increased if AYAs are referred to pediatric and adult specialized cancer centers that offer clinical trials [30].

A major limitation of this review is the lack of studies assessing recruitment and retention strategies. The literature discussing recruitment and retention strategies for AYA cancer patients is less than that for patients of other ages, and not all studies reported their recruitment and retention rates. In two articles [8,9], the authors collected recruitment rates separately based on different strategies without stating their total population, which made it difficult to calculate the accurate overall rates. Additional studies are needed to identify the optimal recruitment and retention strategies for AYA cancer survivors.

Internet-based recruitment strategies are becoming increasingly utilized, followed by hospital outreach and other conventional methods, such as mailing, flyers, and phone calls. Providing monetary incentives is an effective recruitment and retention method in AYA cancer studies. Other retention strategies include frequent email reminders and stable contacts with participants. In future research, evolving communication strategies, such as advertisements on social media (e.g., Facebook, Instagram) and video platforms (e.g., YouTube, Reels, TikTok), can be implemented to improve AYA cancer patient recruitment rates. Investigators should consider cancer survivors’ psychological and social barriers and facilitators to enroll and remain in the studies. There is also an opportunity for future research to address the underlying factor for the low participation rate of AYA cancer patients in cancer clinical trials. More strategies need to be implemented to overcome the retention barriers, such as unwillingness to the time commitment and medical mistrust. It is necessary for investigators to be educated on recruitment and retention barriers faced by AYAs as well as the need to increase education regarding cancer research and treatments for AYAs to improve their knowledge of cancer research and the relationships between patients, healthcare providers, and researchers. Engaging AYA cancer survivors with the research studies they are participating in also may result in higher retention rates.
